# MSC exosomes attenuate sterile inflammation and necroptosis associated with TAK1-pJNK-NFKB mediated cardiomyopathy in diabetic ApoE KO mice

**DOI:** 10.3389/fimmu.2024.1348043

**Published:** 2024-02-08

**Authors:** Abha Banerjee, Dinender K. Singla

**Affiliations:** Division of Metabolic and Cardiovascular Sciences, Burnett School of Biomedical Sciences, College of Medicine, University of Central Florida, Orlando, FL, United States

**Keywords:** inflammation, cell death, diabetes cardiac dysfunction, exosomes, necroinflammation, dyslipidemia

## Abstract

**Introduction:**

Diabetes is a debilitating disease that leads to complications like cardiac dysfunction and heart failure. In this study, we investigated the pathophysiology of diabetes-induced cardiac dysfunction in mice with dyslipidemia. We hypothesize diabetes in ApoE knockout (ApoE-/-) mice induces cardiac dysfunction by increasing inflammation and necroptosis.

**Methods:**

ApoE-/- mice were divided into experimental groups: Control, Streptozotocin (STZ), STZ + MSC-Exo (mesenchymal stem cell-derived exosomes), and STZ+MEF-Exo (Mouse embryonic fibroblast derived exosomes). At Day 42, we assessed cardiac function, collected blood and heart tissues. Heart tissue samples were analyzed for inflammation, necroptosis, signaling mechanism, hypertrophy and adverse structural remodeling using histology, immunohistochemistry, western blotting, RT-PCR, cytokine array and TF array.

**Results and Discussion:**

STZ treated ApoE-/- mice developed diabetes, with significantly (p<0.05) increased blood glucose and body weight loss. These mice developed cardiac dysfunction with significantly (p<0.05) increased left ventricular internal diameter end diastole and end systole, and decreased ejection fraction, and fractional shortening. We found significant (p<0.05) increased expression of inflammatory cytokines TNF- a, IL-6, IL-1a, IL-33 and decreased IL-10 expression. Diabetic mice also exhibited significantly (p<0.05) increased necroptosis marker expression and infiltration of inflammatory monocytes and macrophages. MSC-Exos treated mice showed recovery of diabetes associated pathologies with significantly reduced blood glucose, recovered body weight, increased IL-10 secretion and M2 polarized macrophages in the heart. These mice showed reduced TAK1-pJNK-NFKB inflammation associated expression and improved cardiac function with significantly reduced cardiac hypertrophy and fibrosis compared to diabetic mice. Treatment with MEF-Exos did not play a significant role in attenuating diabetes-induced cardiomyopathy as these treatment mice presented with cardiac dysfunction and underlying pathologies observed in STZ mice.

**Conclusion:**

Thus, we conclude that cardiac dysfunction develops in diabetic ApoE-/- mice, arising from inflammation, necroptosis, and adverse tissue remodeling, which is ameliorated by MSC-Exos, a potential therapeutic for diabetes-induced cardiomyopathy.

## 1 Introduction

Diabetes, a global health emergency, is rapidly expanding in prevalence (1). While the molecular pathology behind the onset of diabetes and its various types are still unclear, chronic systemic metabolic dysfunction, glucose and lipid dysregulation are clear hallmarks of disease which potentiates several complications including cardiomyopathy and cardiovascular disease (1). Poorly controlled type 1 diabetes is associated with elevated lipid levels similar to those with type 2 diabetes (2). In both type 1 and type 2 diabetes, poor glycemic control is observed to impact lipid and lipoprotein metabolism, leading to increased triglycerides and risk of development of cardiovascular disease (CVD) (2). CVD accounts for 60-70% of deaths among diabetic patients(2). Diabetes alone increases risk of heart failure 2-3-fold (3). The dual role of hyperlipidemia and hyperglycemia in inducing cardiomyopathy in type 1 diabetes remains elusive. The present study investigates the pathological mechanisms behind diabetic cardiomyopathy in a type 1 diabetic mouse model with dyslipidemia.

Lipid overload in non-adipose tissues is known to cause cell death (4, 5). Previously our lab reported hypercholesterolemia induces apoptosis and accelerates diabetic complications like cognitive dysfunction (6). Cell death activation in cardiomyocytes is strongly associated with development of cardiomyopathy (7). Cell death and inflammation are potent drivers in disease pathologies that perpetuate each other (8). Apoptosis of myocardial cells in cardiomyopathy has been previously extensively studied. However, under pathological conditions such as heart failure, population of cells undergoing apoptosis compared to other cell death mechanisms are relatively low. In heart failure, it has been reported that significant populations of cells undergo necrosis while only a few succumb to apoptosis (1, 4, 5, 9). Recently, researchers have identified programmed necrosis cell death named necroptosis, as a culprit for many pathological disorders (1, 4, 5, 9). While necroptosis has been observed in numerous pathologies, only recently have a few investigated the role of necroptosis in cardiovascular disease (9). This study identifies for the first time to the best of our knowledge, that necroptosis plays a role in mediating cardiac dysfunction in diabetic hyperlipidemia. Necroptosis is triggered by inflammatory stress stimuli like TNF-α which further activates proteins: RIPK1(receptor-interacting serine/threonine-protein kinase 1) and RIPK3 (receptor-interacting serine/threonine-protein kinase 3), forming the necrosome complex, a large amyloid-like structure which activates MLKL (pseudokinase mixed lineage kinase domain-like protein) to induce membrane rupture and release of inner cell contents which increases inflammation and cell death as a positive feedback loop (8, 10, 11).

Current treatment for type 1 diabetes is lifelong insulin administration. Although this treatment is sufficient for management of disease, it is not a cure and not an effective therapeutic strategy (12). Type 1 diabetic patients face hypoglycemic episodes and devastating complications. Some therapeutic strategies being sought, include pancreatic islet transplantation and stem cell treatment (13). Pancreatic islet transplantation is the most clinically relevant strategy as it provides the means for patients to self-produce insulin. While this therapeutic is sufficient for management of disease, this comes with reliance on immunosuppressant, anti-inflammatory drugs with detrimental side effects (12).

Use of stem cells can circumvent some of these problems, as they have both regenerative and immunomodulating effects. Application of different stem cells: ESC (Embryonic stem cells), iPSCs (induced pluripotent stem cells), and MSCs (mesenchymal stem cells) are promising, however they have tumorigenic potential (14). While ESCs and iPSCs have shown great promise, their use is hindered by controversy, low efficiency, and high cost (14). MSCs are among the most highly studied stem cells, with unique homing features and migration to damaged tissues (13). MSCs have been extensively studied in both diabetic animal models (treated with STZ) and diabetic patients showing remarkable results as a safe and promising therapeutic (12, 13, 15). MSC administration in diabetic models has been observed to reduce blood glucose, elevate insulin levels, reverse insulin resistance, and promote tissue regeneration (12, 13, 15). MSC derived exosomes (MSC-Exo) has recently been shown to bypass tumorigenic potential of stem cells and exhibit higher potency than MSC cells alone in alleviating diabetic pathologies (12). However, it has not yet been investigated, if MSC-Exos can alleviate dyslipidemia and diabetes-induced cardiac dysfunction in a type 1 diabetic model. In the present study, we isolated exosomes from MSCs to evaluate their therapeutic efficacy in mitigating inflammation, necroptosis of cardiomyocytes, and cardiac dysfunction in diabetic ApoE KO mice.

## 2 Methods

### 2.1 Exosome isolation

Exosomes were isolated from Mesenchymal Stem Cells (OriCellTM, C57BL/6 Mouse Bone Mesenchymal Stem Cells; Santa Clara, CA) and Mouse Embryonic Fibroblasts (MEF, ATCC, Manassas, VA). MSC and MEF cells were cultured following supplier’s instructions and grown to 80% confluency, after which growth medium was replaced with knockout DMEM (Dulbecco’s Modified Eagle Medium). Cells were grown in serum free knockout DMEM for 48 hours and subsequently, culture medium was collected, centrifuged, and mixed with ExoQuick solution (System Biosciences, cat# EXOTC50A-1) in 1:5 ratio as suggested in supplier’s protocol. Exosomes from MSC (MSC-Exos) and MEF (MEF-Exos) were isolated following manufacturer’s instruction as published previously by us (16). Following isolation of exosomes, protein concentration was estimated using Bio-Rad protein assay and microplate reader (Bio-Rad, cat# 1681135) was used to determine the absorbance at 595nm. Exosome characterization was performed using western blot for exosome specific markers CD63 and HSP70 as reported by us (17).

### 2.2 Animal model

**Figure 1:**
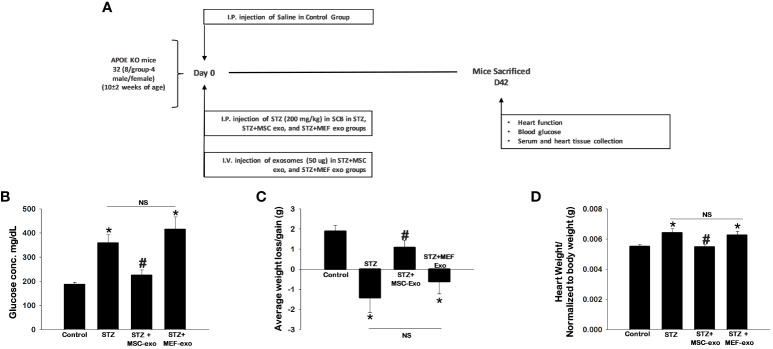
MSC-Exo treatment shows improved blood glucose, body weight and heart weight. **(A)** Study Design Schematic **(B–D)** 42 days post-injection of exosomes, we assessed blood glucose, body weight, and heart weight. Error bars = mean ± standard error of the mean (SEM). *p<0.05 vs. control; #p<0.05 vs. STZ; NS, non-significant; n=5-7; n = number of animals.

Animal studies were conducted in accordance with established and approved protocols of the Institutional Animal Care and Use Committee (IACUC) at the University of Central Florida (UCF). A total of 32 ApoE-/- mice (10 ± 2 weeks of age, equal number of males and females per group) were divided into four groups and were administered the following: Control (Saline), STZ (Streptozotocin: MP Biomedicals, Irvine CA), STZ+MSC-Exos, STZ+MEF-Exos. Single dose of STZ (200 mg/kg body weight) was used to establish a model of type 1 diabetes (**Figure 1A**). STZ was dissolved in sodium citrate buffer and administered by intraperitoneal injection (IP). Fifty µg MSC-Exos and MEF-Exos dissolved in 50 µl KO DMEM were delivered by intravenous (IV) injection. 42-days post-injection (D42), body weight and glucose levels were measured. Change in body weight was examined by calculating the difference in weight prior to receiving injections and at D42. Echocardiography was performed and heart function was analyzed. Next, animals were sacrificed under 4% isoflurane sedation followed by cervical dislocation. Blood was collected by heart puncture. Mice hearts were harvested, washed, weighed, and cut transversely. The top portion of the hearts were stored at -80°C for western blot and RT-PCR studies, while the bottom portions were stored in 4% paraformaldehyde for further histological processing.

### 2.3 Blood glucose measurement

At D42, blood was collected from the tail and a handheld OneTouch Ultra Mini Accu-Check glucose meter (Roche, Indianapolis, IN) device was used to measure blood glucose.

### 2.4 Echocardiography

At D42, echocardiography was performed using Philips Sonos 5500 (Philips, Andover MA). M-mode imaging of the heart was used to determine left ventricular internal diameter end diastole and end systole (LVIDd and LVIDs), ejection fraction (EF%), and fractional shortening (FS). End diastolic volume [EDV; EDV = 7/(2.4+LVIDd) * (LVIDd x 10^3)] and end systolic volume [ESV; ESV = 7/(2.4+LVIDs) * (LVIDs x10^3)] were then calculated. Averages of cardiac parameters (LVIDd, LVIDs, EDV, ESV, EF, FS) were calculated and depicted in bar charts.

### 2.5 Tissue processing

Tissues were processed using Leica TP1020 tissue processing system (Leica Biosystems, Deer Park, IL) and embedded in paraffin using Tissue Tek TEC embedding machine (Sakura, Torrance, CA). 5-micron sections were cut using a microtome and placed on Colorfrost Plus slides.

### 2.6 Immunohistochemistry

Paraffin was removed and tissues were rehydrated. Tissue sections were blocked in 10% goat serum (GS). Sections were stained first with cardiomyocyte specific structural protein α-Sarcomeric actin (1:350, Sigma-Aldrich cat#A2172). Staining for α-Sarcomeric actin was performed using Mouse-on-Mouse kit (Vector Laboratories) following manufacturer’s instructions as reported previously by us (18, 19). In brief, biotinylated anti-mouse IgG, fluorescein Avidin, and Alexa Fluor 488 (1:500; ThermoFisher Scientific cat# A11008) were used to stain cardiomyocytes green. Next, sections were blocked once again in 10% goat serum and then stained for target markers with primary antibodies for: TNF-α (Tumor necrosis factor alpha; 1:200; abcam ab6671), IL-6 (Interleukin-6; 1:200; abcam ab6672), IL-10 (Interleukin-10; 1:200; abcam ab189392), RIPK1 (1:400; sigma SAB3500420), RIPK3 (1:200; sigma PRS2283), MLKL (mixed lineage kinase domain-like protein; 1:200; sigma ZRB1142), IL-1 α (Interleukin 1 alpha; 1:200; abcam ab7632), IL-33 (Interleukin-33; 1:750; abcam ab187060), CD14 (cluster of differentiation 14; 1:200; ABBIOTEC Cat#251561), iNOS (inducible nitric oxide synthase; 1:200; abcam ab15323), Arginase-1 (1:200; Santa Cruz sc-18351), CD206 (cluster of differentiation 206; 1:200; abcam ab64693), pJNK (phosphorylated c-Jun N-terminal kinase; 1:200; Cell Signaling Cat# 4668S), TAK1 (Transforming growth factor-β-activated kinase 1; 1:200; proteintech Cat# 12330-2-AP), NFκB (Nuclear factor kappa B; 1:200; Cell Signaling Cat# 8242S) overnight at 4°C. Sections were then incubated in secondary antibody Alexa Fluor® 568 (1:1000; Life Technologies cat#1700327) for 1 hour at room temperature. Finally, DAPI (4',6-diamidino-2-phenylindole, Vector Labs Cat# #H-1200) containing mounting medium was placed on tissue sections and tissues were imaged using the Keyence BZ-X810 (Keyence, Itasca, IL) microscope. Four images per mouse were used for quantification collected at 20x magnification and representative images were imaged at 40x magnification. Percent expression was calculated by determining the ratio of marker positive cells over DAPI [DAPI (+Marker expression)/DAPI * 100].

### 2.7 Real-time quantitative PCR

TRIzol solubilization and extraction of RNA was performed as previously reported by us (16, 18, 19). In brief, heart tissues stored in -80°C at the time of tissue harvest were processed with TRIzol, chloroform, isopropanol, ethanol, and finally RNA suspension in RNase-free water. SuperScript™ III First-Strand Synthesis SuperMix kit (ThermoFisher; cat# 11752050) was used to perform cDNA synthesis. Amplification and gene expression was performed using CFX96 (Bio-Rad, Hercules, CA) with SYBR Green detection and mouse specific primers (**Supplementary Table 1**). Gene expressions were normalized with Glyceraldehyde 3-phosphate dehydrogenase (GAPDH). Bio-Rad CFX Software was used to determine normalized fold expressions as previously reported by us [16-18].

### 2.8 Western blot

Heart tissues for western blot were prepared in radioimmunoprecipitation assay buffer (RIPA buffer) and tissues were homogenized using a Sonic Dismembrator (Fisher scientific, Waltham MA). Protein concentrations were determined using Bio-Rad Protein Assay Solution (Bio-Rad, cat #5000006) and absorbance was measured by a microplate reader (Bio-Rad, cat# 1681135). 10-25 µg of total protein were run on BoltTM Bis-Tris Mini Protein Gels (ThermoFisher; cat# NW04120BOX) and transferred to polyvinylidene fluoride (PVDF) membranes using the iBlot2 Dry Blotting system (ThermoFisher; cat# 1IB21001) with iBlot2 transfer stacks (ThermoFisher; cat# IB24001). PVDF membranes were then blocked with 5% BSA (Bovine Serum Albumin) or nonfat dry milk dissolved in Tris-buffered saline and Tween-20 (TBS-T) solution. Membranes then were incubated overnight in primary antibodies: RIPK1 (1:400; sigma; SAB3500420), RIPK3 (1:200; sigma; PRS2283), MLKL (1:200; sigma; ZRB1142), CD14 (1:200; ABBIOTEC; Cat#251561), Arginase-1 (1:200; Santa Cruz; sc-18351), TAK1 (1:200; proteintech; Cat# 12330-2-AP), NFκB (1:200; Cell Signaling; Cat# 8242S), and GAPDH (1:1000; Cell Signaling cat#5174S) overnight at 4°C. Horseradish peroxidase (HRP)-linked secondary antibody (1:1000; Cell signaling cat#7074S) incubation was then done at room temperature for one hour. Chemiluminescent detection was then performed using Pico PLUS Chemiluminescent Substrate (ThermoFisher; cat# 34580) and Azure Sapphire Biomolecular Imager [Azure biosystems, Dublin, CA].

### 2.9 Tissue remodeling examined with hematoxylin and eosin and Masson’s trichrome staining

Tissue deparaffinization and rehydration was performed. Sections were then stained as previously reported (16, 18, 19). In brief, H&E staining was done by incubating tissues in: Hematoxylin, 1% Acid Alcohol solution, Bluing agent, Eosin; Masson’s Trichrome Staining was performed by incubating tissues in: Bouins Fixative, Weigert Iron Hematoxylin, Biebrich Scarlet Acid- Fuschin solution, phosphomolybdic/Phosphotungstic Acid solution, Aniline Blue solution, and 1% Glacial Acetic acid. Tissues were then dehydrated and Permount™ (Fisher Scientific; cat# SP15-500) mounting medium was used to preserve stained tissues. Keyence BZ-X810 (Keyence, Itasca, IL) was used to acquire tissue images. Images collected at 20x magnification were used for quantification and images taken at 40x were used for representative figures. Differences in myofibril size, to determine tissue hypertrophy, was determined from H&E-stained sections, and the size of blue fibrotic areas were determined from Masson’s trichrome stained slides. Images were analyzed and size areas were collected using Image J software.

### 2.10 Cytokine array

RayBio® C-Series Mouse Cytokine Antibody Array 1000 (RayBiotech, Cat# AAM-CYT-1000-2) was used to assess differential expression of cytokines. Heart tissue homogenates were prepared in PBS and used to perform the assay following the manufacture’s protocol. Blots were imaged using the Azure Sapphire Biomolecular Imager [Azure biosystems, Dublin, CA] and image J was used to perform densitometric analysis.

### 2.11 Transcription factor array

We used the Cholesterol Metabolism TF Activation Profiling Plate Array (Signosis, Cat# FA-1008) to survey differential cholesterol regulation and metabolism. Nuclear Extraction kit (Signosis, Cat# SK-0001) was used to prepare nuclear extracts from 20 mg heart tissues. Profiling of cholesterol transcription factors was done following manufacturer’s instructions. In brief, 10 µg of nuclear extract was incubated with biotin labelled probes. Bound TF/probe complexes were then isolated, eluted, and hybridized with oligos in a pre-coated plate. Conjugated complexes were then detected with Streptavidin-HRP Conjugate and luminescence was detected by a microplate luminometer (SpectraMax® i3x (Molecular Devices, San Jose, CA).

### 2.12 Data analysis

Statistical significance was determined by Two-tailed Student’s t-test and One-way analysis of variance (ANOVA) followed by Tukey test. Sigma Plot software was used to perform statistical analysis and generate data histograms with values presented as ± standard error mean (SEM). Statistical significance of p<0.05 was used.

## 3 Results

### 3.1 MSC-Exosomes mitigate hyperglycemia and weight loss, potentiated by STZ in ApoE KO mice

Our model of diabetic apolipoprotein E knockout (ApoE KO) mice and exosome treatment groups were established as depicted in **Figure 1A**. STZ antagonized glucose levels in ApoE KO mice with significantly (p<0.05) increased blood glucose (**Figure 1B**). Additionally, significantly reduced body weights of ApoE KO mice were observed upon STZ injection (**Figure 1C**). Furthermore, we measured heart weights normalized to body weights of the mice, and we found significantly (p<0.05) increased heart weights along with metabolic alterations, implicative of changes in tissue pathology of the heart, in diabetic (STZ administered) ApoE KO mice compared to control (**Figure 1D**).

These hallmarks of diabetes were found to be ameliorated by MSC-Exos treatment. Significantly (p<0.05) reduced glucose levels and retained body weights were observed in MSC-Exo treated mice compared to the STZ administered group. Additionally, these MSC-Exo treated mice exhibited significantly reduced heart weights (p<0.05) compared to STZ mice. MEF exosome (MEF-Exos) treatment did not attenuate disease phenotypes, as these mice had elevated blood glucose, reduced body weight, and increased heart weights compared to control. This provides evidence that exosomes derived from MSCs specifically ameliorates diabetes pathologies where MEF exosomes fail to do so.

### 3.2 MSC-exosomes reduce pro-inflammatory factors secreted by immune cells: TNF-α and IL-6 and upregulate anti-inflammatory cytokine IL-10 in diabetic ApoE KO mice

**Figure 2:**
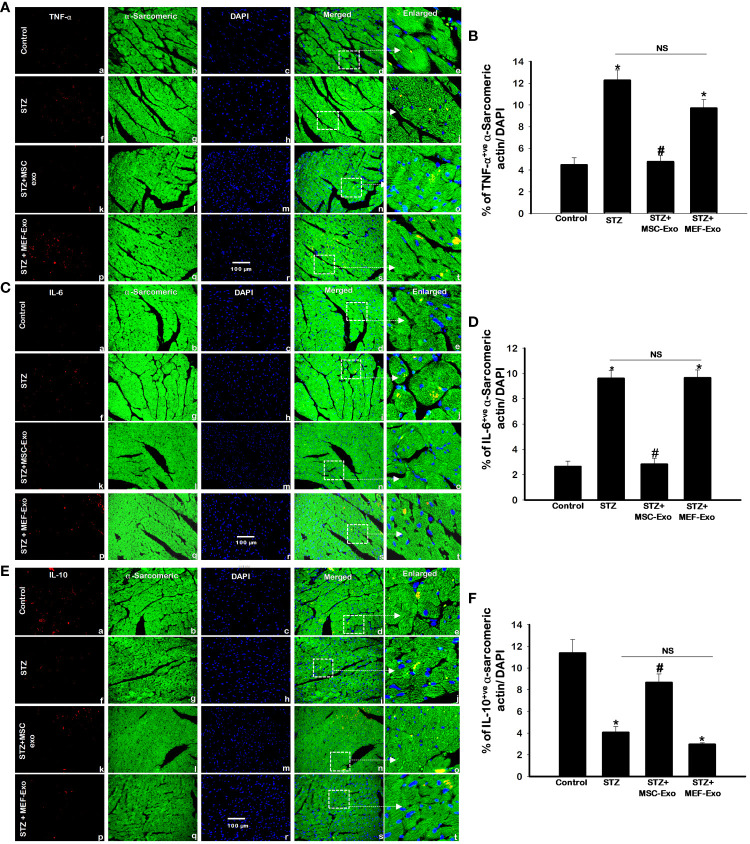
MSC-Exo treatment reduced pro-inflammatory TNF-α and IL-6 and increased IL-10 levels in the heart. Fluorescent detection of TNF-α, IL-6, IL-10 are shown in panel **(A, C, E)** respectively. Quantification is shown in panel **(B, D, F)**. IHC images of staining for TNF-α, IL-6, IL-10 labelled in red (a, f, k, p); α-sarcomeric actin in green (b, g, l, q); nuclear DAPI stained in blue **(c, h, m, r)**. Merged images are shown in panels d, i, n, s and enlarged images in (e, j, o, t). White boxes and arrows identify the areas depicted in enlarged images. Error bars = mean ± standard error of the mean (SEM). Scale bar = 100 μm. *p<0.05 vs. control; #p<0.05 vs. STZ; NS, non-significant; n=8 mice. n = number of animals.

Cytokine expression of TNF-α, IL-6, and IL-10 were measured by immunohistochemistry (IHC). Representative IHC images for TNF- α, IL-6 and IL-10 are shown in **Figures 2A, C, E** respectively, and quantification of detected cytokine positive cardiomyocytes are depicted in **Figures 2B, D, F**.

STZ administered diabetic ApoE KO mice compared to control exhibited significantly (p<0.05) increased pro-inflammatory cytokines TNF- α and IL-6 (**Figures 2A–D**). The anti-inflammatory cytokine IL-10, in contrast was significantly (p<0.05) downregulated in the STZ group when compared to control (**Figures 2E, F**). The release of inflammatory TNF-α corresponds to inflammatory monocyte and M1 macrophage secretion whereas IL-10 is secreted by M2 macrophages as published by us and others (18, 20). MSC-Exo treated mice resulted in significant (p<0.05) reduction in pro-inflammatory TNF-α and IL-6, while IL-10 levels were increased when compared to STZ group. This finding indicates MSC-Exos may be providing its therapeutic role by supporting an anti-inflammatory milieu by resident cells like immune cells in the myocardium of diabetic ApoE KO mice. Moreover, MEF-Exo treated mice showed significantly (p<0.05) increased pro-inflammatory cytokines (TNF-α, IL-6) and decreased anti-inflammatory IL-10 when compared to control, suggesting MEF-Exo treatment is insufficient in constraining pro-inflammatory cytokine expression in diabetic ApoE KO mice.

### 3.3 MSC-exosome treatment reduces expression of necroptosis markers in diabetic ApoE KO mice

**Figure 3:**
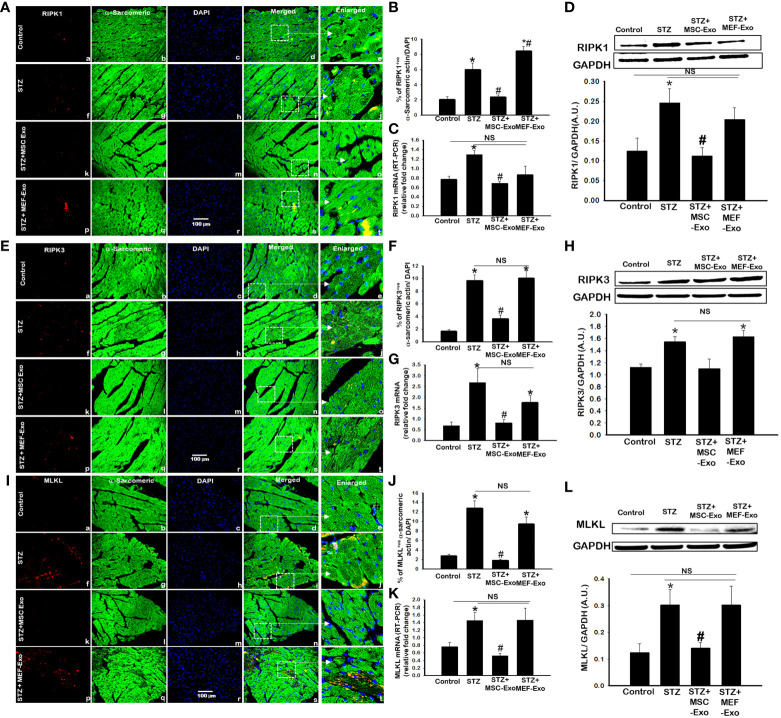
MSC-Exo treatment reduced inflammatory necroptosis cell death in the heart. **(A, E, I)** Representative immunohistochemical images of staining for RIPK1, RIPK3, and MLKL labelled in red (a, f, k, p); α-sarcomeric actin in green (b, g, l, q); nuclear DAPI stained in blue (c, h, m, r). Merged images are shown in panels d, i, n, s and enlarged images in panels e, j, o, t. White boxes and arrows identify the areas depicted in enlarged images. Scale bar = 100 μm. **(B, F, J)** Quantifications of immunohistochemistry; **(C, G, K)** RT-PCR gene expression; **(D, H, L)** western blots. Error bars = mean ± standard error of the mean (SEM). *p<0.05 vs. control; #p<0.05 vs. STZ; NS, non-significant; n=5-8 mice. n = number of animals.

Inflammatory cell death pathway necroptosis was investigated as a possible mechanism for the pathology of cardiomyopathy. Necroptosis proteins RIPK1, RIPK3, and MLKL expression was measured using IHC, PCR, and western blot. Overall, we found upregulation of necroptosis cell death proteins in the STZ group when compared to control. At both gene and protein expression levels, necroptosis markers: RIPK1 (**Figures 3A–D**), RIPK3 (**Figures 3E–H**, and MLKL (**Figures 3I–L**) were significantly (p<0.05) increased when compared to control.

Treatment with MSC-Exos resulted in reduced expression of necroptosis proteins. Significant (p<0.05) decrease in RIPK1 (**Figures 3A, B**), RIPK3 (**Figures 3E, F**), and MLKL (Figures 3 I, J) was detected by IHC. This was further corroborated by western blot. In **Figures 3D** and **L**, RIPK1 and MLKL showed significantly decreased expression when compared to control. Although western blot analysis of RIPK3 levels in the MSC treated group were not found statistically significant, the trend for its reduced expression was observed (**Figure 3H**). At the gene level, significantly (p<0.05) reduced mRNA transcripts of RIPK1, RIPK3, and MLKL were detected in MSC treated mice compared to STZ mice. Molecular data provides strong evidence that MSC exosomes interfere with the activation of necroptosis cell death of cardiomyocytes by STZ and protect cardiomyocytes from inflammatory cell death activation. Alternatively, MEF-Exo treated mice showed increased expression of necroptosis markers compared to control and non-significant change compared to diabetic mice, suggesting MEF-exosomes did not support downregulation of necroptosis in diabetic ApoE KO mice whereas significantly reduced expression was observed with MSC-Exo treatment.

### 3.4 MSC-Exos treatment attenuates necroinflammation mediated by IL-1α and IL-33 in diabetic ApoE KO mice

**Figure 4:**
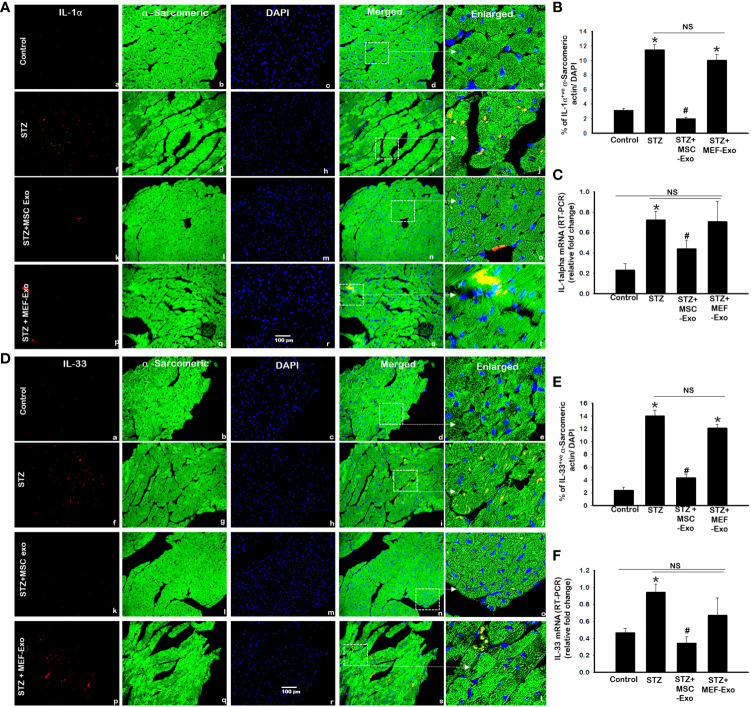
MSC-Exo treatment reduced inflammatory necroinflammation IL-1α and IL-33. Fluorescent detection of IL-1α and IL-33 is shown in **(A, D)**. Quantification is shown in **(B, E)**. IHC images of staining for IL-1α and IL-33 labelled in red (a, f, k, p); α-sarcomeric actin in green (b, g, l, q); nuclear DAPI stained in blue (c, h, m, r). Merged images are shown in panels d, i, n, s and enlarged images in (e, j, o, t). White boxes and arrows identify the areas depicted in enlarged images. **(C, F)** RT-PCR for IL-1α and IL-33 gene expression; Error bars = mean ± standard error of the mean (SEM). Scale bar = 100 μm. *p<0.05 vs. control; #p<0.05 vs. STZ; NS, non-significant; n=6-8 mice. n = number of animals.

IL-1α and IL-33, downstream markers of necroptosis were measured by IHC and RT-PCR. As shown by representative **Figures 4A** and **D** with quantification **Figures 4C** and **F** we found the percentage of cardiomyocytes expressing IL-1α and IL-33 to be significantly (p<0.05) increased in ApoE KO STZ mice compared to control. This was further corroborated by RT-PCR, at the gene level we detected significantly (p<0.05) increased mRNA transcripts of IL-1α and IL-33 in STZ mice when compared to control mice. These data confirm upregulated execution of necroptosis cell death of cardiomyocytes in ApoE KO mice under diabetic conditions. Upon MSC-Exo treatment, mice showed significantly (p<0.05) lowered expression levels of IL-1α and IL-33 at the protein (**Figures 4A, B, D, E**) and gene level (**Figures 4C, F**) when compared to STZ mice. This further suggests that MSC-Exos as an effective therapeutic in hindering STZ-induced necroptosis of cardiomyocytes in diabetic ApoE KO mice. MEF-Exo treated group showed non-significant decrease in IL-1α and IL-33 levels when compared to diabetic mice, an indication exosome therapeutic is cell origin specific.

### 3.5 MSC-Exo treatment inhibits diabetes induced inflammatory monocytes and macrophages in ApoE KO mice hearts

**Figure 5:**
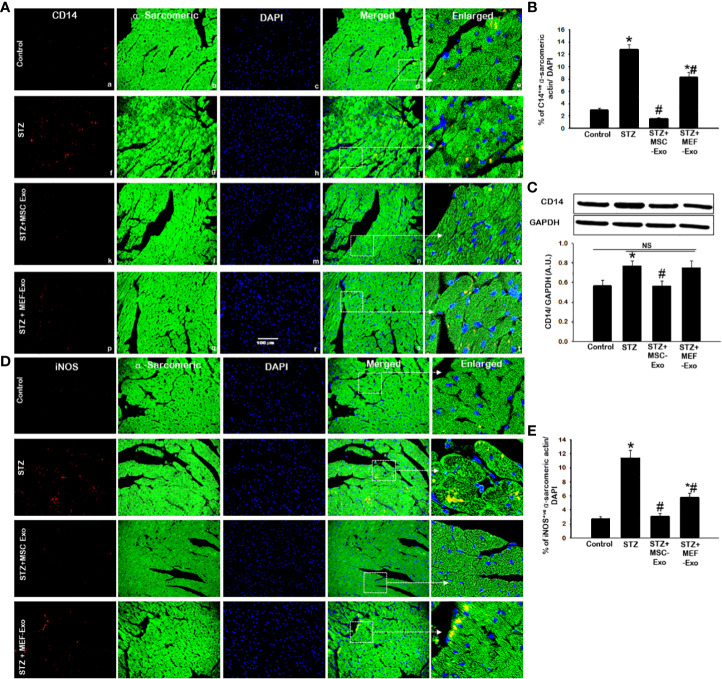
MSC-Exo treatment reduced inflammatory monocyte and macrophage markers CD14, iNOS in the heart. Fluorescent detection of CD14 and iNOS are shown in **(A, D)** respectively. Quantification is shown in **(B, E)**. IHC images of staining for CD14, iNOS labelled in red (a, f, k, p); α-sarcomeric actin in green (b, g, l, q); nuclear DAPI stained in blue (c, h, m, r). Merged images are shown in panels d, i, n, s and enlarged images in (e, j, o, t). White boxes and arrows identify the areas depicted in enlarged images. **(C)** CD14 western blot. Error bars = mean ± standard error of the mean (SEM). Scale bar = 100 μm. *p<0.05 vs. control; #p<0.05 vs. STZ; NS, non-significant; n=5-8 mice. n = number of animals.

In response to cytokine stimuli, infiltration of inflammatory monocytes and macrophages potentiate tissue inflammation. This was investigated by detection of expression of CD14 and iNOS, markers of monocytes and tissue macrophages by IHC and western blot. In diabetic ApoE KO mice, we found increased inflammatory monocyte and macrophage infiltration detected by CD14 (**Figure 5A**) and iNOS marker expression (**Figure 5D**) compared to control mice. Quantification of CD14 and iNOS in the heart tissue of STZ mice revealed, significantly (p<0.05) increased percentage of CD14 and iNOS positive signal compared to control. This was further corroborated by western blot, in STZ mice we found significantly (p<0.05) increased CD14 expression (**Figure 5C**) when compared to control.

MSC-Exo treated mice demonstrated reduced infiltration of inflammatory monocytes and macrophages in the heart. This was evidenced by significant (p<0.05) depletion of CD14 and iNOS positive cells in the heart of MSC-Exo treated mice compared to STZ mice, as depicted in **Figures 5A, D** and quantified **Figures 5B, E**. Western blot analysis further validates significant (p<0.05) reduction of CD14 expression in heart tissue of MSC treated mice when compared to STZ group. These results provide strong evidence for the reduced presence of inflammatory macrophages in MSC-Exo treated mice.

### 3.6 MSC treated diabetic ApoE KO mice exhibit increased M2 anti-inflammatory macrophage presence in the heart

**Figure 6:**
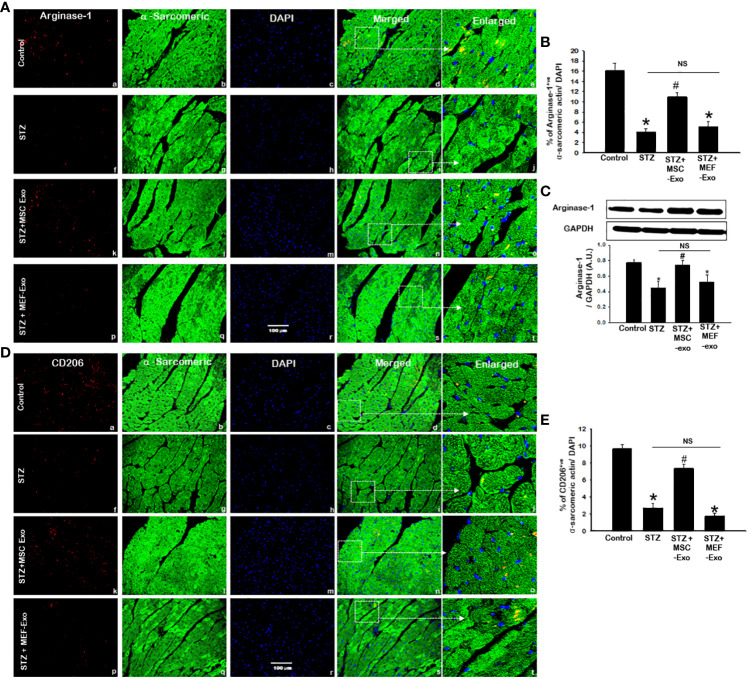
MSC-Exo treatment reduced M2 markers Arginase-1 and CD206 in the heart. Fluorescent detection of Arginase-1 and CD206 are shown in **(A, D)** respectively. Quantification is shown in **(B, E)**. IHC images of staining for Arginase-1 and CD206 labelled in red (a, f, k, p); α-sarcomeric actin in green (b, g, l, q); nuclear DAPI stained in blue (c, h, m, r). Merged images are shown in panels d, i, n, s and enlarged images in (e, j, o, t). White boxes and arrows identify the areas depicted in enlarged images. **(C)** Arginase-1 western blot. Error bars = mean ± standard error of the mean (SEM). Scale bar = 100 μm. *p<0.05 vs. control; #p<0.05 vs. STZ; NS, non-significant; n=8 mice. n = number of animals.

We investigated M2 polarization of macrophages, associated with wound healing and tissue regeneration. To do this we measured arginase-1 and CD206 expression by IHC and western blot. IHC detection of arginase-1 (**Figure 6A**) and CD206 (**Figure 6D**) revealed diminished presence of M2 macrophages in STZ mice compared to control. In STZ hearts, significantly (p<0.05) reduced Arginase-1 and CD206 marker expression was detected by IHC when compared to control (**Figures 6B, E**). We confirmed this finding by performing western blot to detect Arginase-1 in heart tissues and found significantly(p<0.05) reduced Arginase-1 protein levels in STZ mice compared to control. These data suggest tissue macrophages in the heart of STZ mice, likely polarize to M1, propagated by pro-inflammatory cytokine stimuli released from activation of necroptosis cell death of cardiomyocytes.

Increased arginase-1 and CD206 signal was detected in the myocardium of MSC treated mice (**Figures 6A, D**). IHC quantification of arginase-1 and CD206 signal showed significantly (p<0.05) increased arginase-1 and CD206 in MSC-Exo group compared to STZ (**Figures 6B, E**). Moreover, this was supported by western blot analysis of arginase-1 protein expression, which was found to be significantly (p<0.05) increased in MSC-Exo group compared to STZ. This suggests MSC-Exo mice display increased tissue infiltrating M2 macrophages in the heart, which may provide regenerative support under diabetic conditions. MEF-Exo treatment did not increase M2 polarization in the heart, as indicated by reduced levels of arginase-1 and CD206, levels coinciding with STZ mice (**Figure 6**).

### 3.7 MSC Exo treatment in diabetic ApoE KO mice show reduced hypertrophy and fibrosis

**Figure 7:**
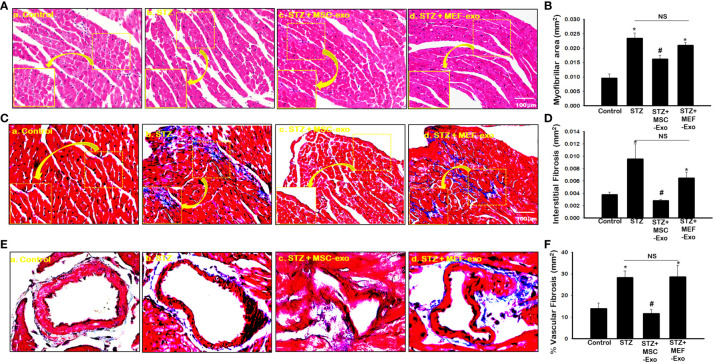
MSC-Exo treatment shows reduced heart atrophy and fibrosis in diabetic ApoE KO mice. **(A)** Hematoxylin and eosin-stained sections of the heart; 40x magnification. **(B)** Myofibrillar area quantified from 4-5 images of stained sections: 20x magnification. **(C–E)** Interstitial Fibrosis (IF) and Intravascular Fibrosis (IV) representative images; 40x magnification. **(D–F)** IF and IV quantification from 4-5 images of stained sections; 20x magnification. Error bars = mean ± standard error of the mean (SEM). *p<0.05 vs. control; #p<0.05 vs. STZ; NS, non-significant; n=6-8; n = number of animals.

Diabetes is well documented to promote cardiovascular disease through adverse tissue remodeling of the heart. We investigated these pathologies by measuring myofibrillar area of cardiomyocytes for signs of hypertrophy, interstitial fibrosis, and vascular fibrosis, indicators of tissue damage. As predicted, we found significantly (p<0.05) increased size of cardiomyocytes in STZ ApoE KO mice (**Figures 7A, B**) when compared to control. Moreover, significantly (p<0.05) increased fibrotic areas within the myocardium and vasculature of the heart was detected by Masson’s trichrome staining in ApoE KO mice compared to the control group (**Figures 7C–F**). Administration of MSC-Exos was found to attenuate these markers of adverse tissue remodeling. Significantly (p<0.05) reduced myofibrillar area of cardiomyocytes was detected in the hearts of MSC-Exo treated mice (**Figure 7B**). Furthermore, we detected significantly (p<0.05) reduced fibrosis within the tissue and vessels of the hearts of MSC-Exo mice when compared to control (**Figures 7C–F**). This provides strong evidence for the therapeutic efficacy of MSC-Exos in mitigating pathology associated with diabetic cardiomyopathy in ApoE KO mice. Treatment with MEF-Exos however was not observed to influence these features of adverse tissue remodeling. MEF-Exo mice were found to exhibit non-significant change in myofibril area and fibrosis when compared to STZ mice, with pathology of the tissue resembling STZ mice (**Figure 7**).

### 3.8 MSC Exo treated diabetic ApoE KO mice show reduced pJNK, TAK1, and NFKB expression

**Figure 8:**
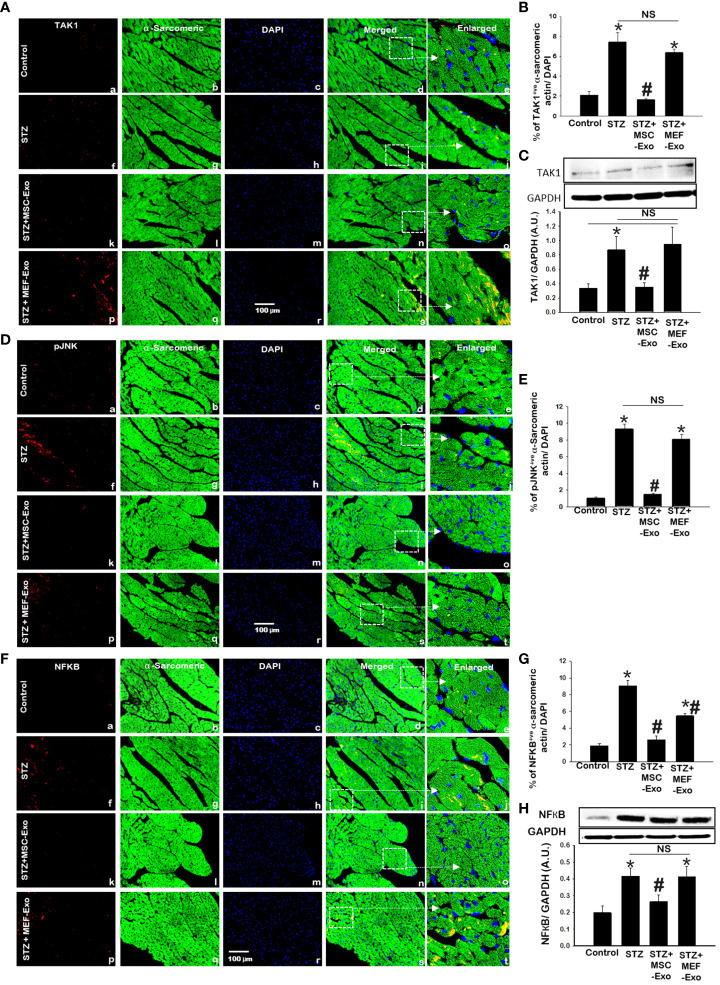
MSC-Exo treatment reduced inflammatory TAK1, pJNK, NFKB expression in the heart. **(A, D, F)** Representative immunohistochemical images of staining for TAK1, pJNK, NFKB labelled in red (a, f, k, p); α-sarcomeric actin in green (b, g, l, q); nuclear DAPI stained in blue (c, h, m, r). Merged images are shown in panels d, i, n, s and enlarged images in panels e, j, o, t. White boxes and arrows identify the areas depicted in enlarged images. Scale bar = 100 μm. **(B, E, G)** Quantifications of immunohistochemistry; **(C, H)** western blots. Error bars = mean ± standard error of the mean (SEM). *p<0.05 vs. control; #p<0.05 vs. STZ; NS, non-significant; n=5-8 mice. n = number of animals.

TAK1, pJNK, NFKB was further explored by IHC and TAK1 and NFKB by western blot. TAK1, pJNK, and NFKB proteins were detected by IHC as depicted in **Figures 8A**, **D**, and **F**. Quantification of marker expression detected in cardiomyocytes are represented in **Figures 8B**, **E**, and **G**. To strengthen our findings, we confirmed our observations by western blot analysis (**Figure 8C, H**). IHC and western blot data showed significantly (p<0.05) increased expression of TAK1 and NFKB proteins in STZ group compared to control (**Figure 8**). In contrast, MSC treated mice led to significantly (p<0.05) decreased expression of TAK1 and NFKB levels when compared to STZ mice. This suggests that MSC-Exos may be playing a role in the heart by reducing TAK1, pJNK, NFKB expression levels. Expression levels of TAK1, pJNK, and NFKB was not reduced by MEF-Exo treatment when compared to control.

### 3.9 MSC Exo treatment improves cardiac function in diabetic ApoE KO mice

**Figure 9:**
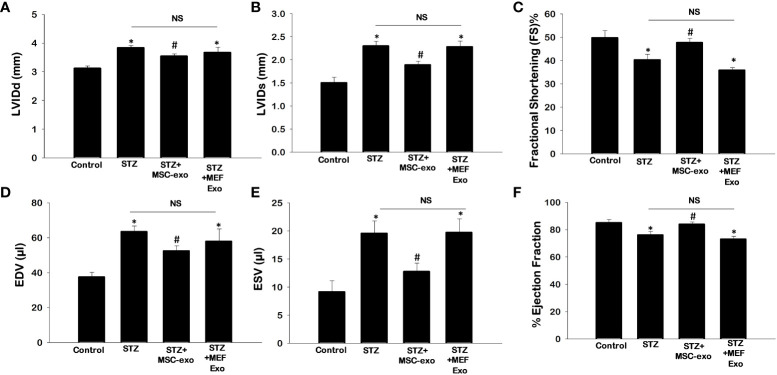
MSC-Exo treatment improves cardiac function. Echocardiography was performed to determine heart function. **(A)** Left ventricular internal dimension-diastole (LVIDd) **(B)** Left Ventricular internal dimension-systole (LVIDs) **(C)** Fractional Shortening [FS%; (LVIDd – LVIDs)/LVIDd × 100)] **(D)** Left Ventricular Volume at End Diastole (EDV) **(E)** Left Ventricular Volume at End Systole (ESV) **(F)** Ejection Fraction (EDV– ESV)/EDV × 100). Error bars = mean ± standard error of the mean (SEM). *p<0.05 vs. control; #p<0.05 vs. STZ; NS, non-significant; n=5-7 mice. n = number of animals.

To determine the effect of diabetic hyperlipidemia on the heart, we measured cardiovascular parameters LVIDd, LVIDs, EDV, ESV, FS, and EF using echocardiography (**Figure 9**). As expected, STZ mice exhibited impaired cardiac function, with significantly (p<0.05) increased LVIDd, LVIDs, EDV, and ESV and decreased EF, and FS when compared to control. Moreover, treatment with MSC-Exo was able to improve cardiac function. Heart function parameters were similar to control where LVIDd, LVIDs, EDV, and ESV were significantly (p<0.05) decreased and there was a significant increase in EF, and FS when compared to STZ mice (**Figures 9A–F**). Significant improvements in cardiac function was not observed in the MEF-Exo treated mice.

### 3.10 MSC exosome treatment in diabetic ApoE KO mice have reduced pro-inflammatory cytokine regulation

**Figure 10:**
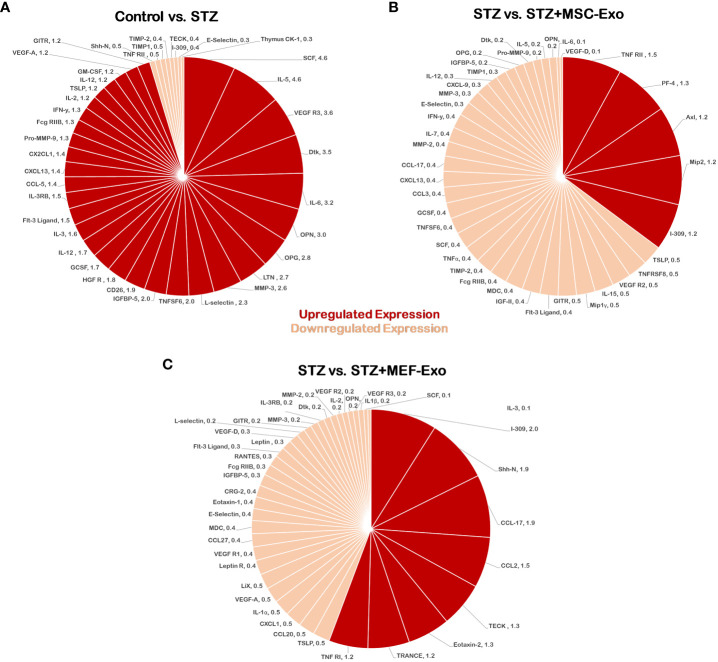
MSC-Exo promotes reduction of pro-inflammatory cytokines. Molecular arrays for inflammatory cytokines were performed. Upregulated cytokines (ratio ≥1.2) are shown in bright red and downregulated (ratio ≤ 0.5) cytokines in light red or pink color; n=1 (single array). **(A)** Control versus STZ mice **(B)** STZ versus STZ+MSC-Exo **(C)** STZ versus STZ+MEF-Exo; n = number of animals.

Differential expression of 96-cytokines in the heart was determined by calculating ratios of relative expression between groups (**Figures 10A–C**). A ratio greater than or equal to 1.2-fold was determined to indicate upregulated expression, while a ratio of less than or equal to 0.5 fold was used to determine downregulation. STZ mice showed upregulated pro-inflammatory cytokine expression. Comparison of control mice versus the diabetic STZ group showed upregulated 31- pro-inflammatory cytokines and downregulated 8-cytokines (**Figure 10A**). In contrast, comparison between STZ group and MSC-Exo treated mice downregulation of 34-proinflammatory cytokines and upregulation of 5-cytokines (**Figure 10B**). This indicates MSC-Exo treatment led to downregulation of inflammatory cytokines. MEF-Exo treated mice also showed reduced levels of pro-inflammatory cytokines. When STZ mice were compared to MEF-Exo treated mice, there was a downregulation of 32-cytokines, and upregulation of 8-cytokines (**Figure 10C**).

Next, we analyzed cholesterol transcription factor activity to investigate cholesterol metabolism in diabetic ApoE KO mice (**Supplementary Figure 1**). Diabetic dyslipidemic conditions were observed to affect cholesterol transcriptional activity of SREBP (Sterol regulatory element binding protein), LXR (liver X receptor), C/EBP (CCAAT/enhancer binding proteins), PPAR (Peroxisome proliferator-activated receptor), COUP-TF (Chicken ovalbumin upstream promoter transcription factor), Nur77 (nuclear receptor 77), and RXR (retinoid x receptor) which has been well documented to be involved in maintaining lipid and carbohydrate homeostasis (21–27).

Reduced activity of cholesterol transcription factors was observed in diabetic mice compared to control mice. Insulin insufficiency in diabetic mice may account for reduced cholesterol transcription factor activity, as these transcription factors are regulated by insulin (21–27).

In contrast, MSC-Exo treated mice exhibited increased cholesterol transcription factor activity, suggesting an ameliorating role of MSC-Exos in metabolic regulation. MEF-Exo treated mice also showed an increase in cholesterol transcription factor activity compared to diabetic mice, however the increase trend was more prominent in the MSC-Exo treated group.

## 4 Discussion

Heart Failure is the most common complication of diabetes (28). The cardiovascular system is one of the most vulnerable organ systems to be affected by diabetes, a major cause for diabetes related morbidity and mortality (1). In diabetes, glucose utilization is compromised, and the heart becomes heavily dependent on fatty acid utilization (29). Excess fatty acid oxidation increases cardiotoxic intermediates and ROS. Lipid dysregulation impairs cardiac function, triggers inflammatory pathways, and promotes cardiomyocyte cell death (4, 5, 29). In the present study, we investigate the pathology and molecular mechanisms underlying cardiac dysfunction induced by diabetes and dyslipidemia in ApoE KO mice. For the first time to the best of our knowledge, we provide evidence for cardiac dysfunction from diabetes in ApoE KO mice, occurring through inflammatory mechanisms and upregulation of necroptosis cell death. We further utilized mesenchymal stem cell derived exosomes (MSC-Exos) to mitigate cardiac dysfunction.

The occurrence of diabetes in ApoE KO mice was confirmed by increased blood glucose levels and decreased body weights post STZ injection. An increase in heart weights were found in STZ administered diabetic mice compared to control, suggesting the occurrence of cardiac hypertrophy due to diabetes. Cardiac hypertrophy is known to prelude heart failure and CVD induced mortality in diabetes (4, 5).

Cytokines and chemokines of stress induced tissues, cause systemic chronic inflammation and disease progression (4, 5, 9). Among these factors, TNFα and IL-6 are well documented to be highly immunogenic and critical in the activation of necroptosis (10, 11, 15, 29–33). As such, in our ApoE KO mouse model, we found an upregulation of TNFα and IL-6 in dyslipidemic mice exacerbated by diabetes. In contrast, anti-inflammatory IL-10 levels were significantly low in diabetic mice compared to control. Thus, concluding a pro-inflammatory shift in cytokine expression in diabetic ApoE KO mice.

Others have reported increased levels of pro-inflammatory cytokines induce necroptosis of myocardial cells increasing risk of heart failure development (4, 5, 28). Cell death and inflammation potentiate disease by perpetuating each other as a positive feedback loop (8). Loss of cardiomyocytes is an important factor in the progression of heart failure (28, 31). Necroptosis, a novel form of inflammatory cell death has been observed in numerous pathologies, and only recently has been studied in CVD. In our investigation of the hearts of diabetic ApoE KO mice, we found highly expressed necroptosis mediators (RIPK1, RIPK3, MLKL) and proceeding downstream necroinflammation (IL-1α, and IL-33) compared to control. This overactivation of necroptosis, is highly culpable in inducing cardiac tissue damage, previously identified in other disease pathologies to progress to heart failure (9). Sustained chronic inflammation is well documented to proceed to increased leukocyte tissue infiltration with corresponding exaggerated proinflammatory cytokine secretion and accelerated cardiac tissue remodeling in diabetes (32, 34).

Majority of leukocytes in the heart are macrophages (35). Upon injury, macrophage population in the heart is further increased by infiltration of circulating monocytes (35). Infiltrating monocytes and resident macrophages activate upon contact with DAMPs (damage associated molecular patterns) released by inflammatory cell death like necroptosis (35).

Macrophages central in CVD development, rapidly respond to stimuli and play adaptive role in promoting inflammation (M1 macrophage polarization) and wound healing (M2 polarization) (30). Macrophages facilitate cardiac remodeling and interstitial inflammation regulation. Dysregulation of macrophages has been observed in inflammation mediated cardiac injury. As such, macrophage polarization and sub population balance, is important in tissue regeneration and homeostasis (36). M1 macrophages sustain inflammation necessary for clearance of pathogens, activated by TNF-α, IL-1α, and IL-6 (30, 35, 36). Overactivation of M1 macrophages has been identified in age related diseases, leading to tissue damage and impaired wound healing (30, 35, 36). In contrast, M2 macrophage polarization, induced by IL-10, promotes tissue repair and wound healing by stimulating angiogenesis and dampening inflammation (30). Macrophage sub-population characterization by identification of cell surface markers for inflammatory macrophages and monocytes (CD14, iNOS) and M2 macrophages (Arginase-1, CD206) were examined in the hearts of diabetic dyslipidemic mice as done previously by us and others and presented in this study (20, 30). Previous reports have identified inflammatory monocyte and macrophage upregulation to precede cardiac dysfunction in diabetes, causing chronic inflammation and insulin resistance (34). Our results coincide with these reports, as we saw increased expression of inflammatory monocyte and macrophage markers in our diabetic ApoE KO model. The release of inflammatory TNF-α corresponds to inflammatory monocyte and M1 macrophage secretion whereas IL-10 is secreted by M2 macrophages as published by us and others (18, 20). Inflammatory factors IL-6 and TNF-α expression can be attributed to the increase in tissue infiltrating inflammatory monocytes and macrophages detected in the myocardium of diabetic mice. Further, low levels of M2 macrophage were detected in diabetic mice compared to control and may account for the reduced IL-10 levels we detected in diabetic mice compared to control.

Further, we evaluated morphological features of cardiac damage, hypertrophy and fibrosis, known to lead to cardiac impairment (1). The occurrence of hypertrophy in diabetic ApoE KO mice was confirmed, as we found marked increase in myofibril size of cardiomyocytes compared to non-diabetic ApoE KO mice. Chronic myocardial inflammation in diabetes mediates tissue structure damage, and hypertrophy which can result in heart failure. Inflammation plays a crucial role in the pathology of diabetes-induced cardiac hypertrophy (29). This correlates with our present study as we detected increased areas of fibrosis within the tissue and around blood vessels in the heart of diabetic ApoE KO mice. We provide strong evidence that pro-inflammatory cytokines, upregulated necroptosis cell death, and increased tissue infiltrating inflammatory monocytes and macrophages in the heart delineating adverse cardiac remodeling, exacerbating diabetes-induced cardiac dysfunction in ApoE KO mice.

Systemic inflammation is mediated by various cytokines, have a common endpoint, activation of NFKB. Increased expression of NFKB is well documented in cytokine induced myocardial and vascular damage (29). High glucose has been previously shown to increase TAK1 expression and subsequent inflammatory responses from activated TAK1-JNK-NFKB signaling (37). In necroptosis, TNF-α stimulation promotes activation and RIPK1 ubiquitination with subsequent necrosome formation and recruitment of TAK1 and JNK which acts on NFKB transcription (10, 11, 37). Therefore, in the present study we examined TAK1, pJNK, NFKB expression for mediation of myocardial inflammation and adverse tissue remodeling in diabetic ApoE KO mice. We detected significantly increased protein expression levels for TAK1, pJNK, and NFKB in the hearts of diabetic mice compared to control. Thus, confirming increased TAK1, pJNK, NFKB expression a potential mechanism in diabetes induced intracardial inflammation and cardiac injury.

Increased inflammatory stimuli induces myocardial fibrosis by enhanced cardiac fibroblast proliferation, which deposits collagen resulting in myocardial stiffness and contractile dysfunction (29). Therefore, in our study of ApoE KO diabetic mice, we found impaired cardiac function indicated by increased systolic and diastolic functions (LVIDd, LVIDs, EDV, and ESV) and reduced ejection fraction and fractional shortening.

Strategies to delay onset of cardiac hypertrophy and cardiac inflammation has been a potential mechanism in reducing CVD induced mortality in diabetic patients (1, 4, 5, 9, 28). The use of Mesenchymal stem cells has recently gained traction in the treatment of diabetic patients (15). Diabetic patients treated with MSCs have shown to respond with stable blood glucose levels, with some becoming independent or receive low dose insulin to maintain blood glucose levels (15). However, several disadvantages of MSC cell therapy have been reported, as MSCs have tumorigenic potential, cause thrombosis and fever, as well as exhibit low survival time within the body. Paracrine action of MSCs have been observed to provide its regenerative effect. Further investigations have revealed exosomes as the mediator for this (15). MSCs produce immunoregulatory exosomes and have a significant impact in immunomodulation (36). Recently, it has been shown, MSC-Exos have greater regenerative capabilities than MSC cells themselves (12, 15).

The IV injection of exosomes has demonstrated effects on the heart in reducing apoptosis, pyroptosis, inflammation, cardiac hypertrophy with improved cardiac function as reported by various investigators (15, 16, 34, 36). Therefore, our current investigation is in agreement with reduced necroptosis, inflammation, and improved cardiac function following exosome treatment in ApoE knockout diabetic mice. In this present study, we utilized MSC-Exo treatment and investigated its potential in attenuating diabetes induced cardiac injury. Here we present novel evidence for the use of MSC-Exo treatment in inhibiting inflammation and attenuating cardiac damage in diabetic ApoE KO mice. In this study, we found treatment of MSC-Exos in STZ treated ApoE KO mice constrained diabetes pathologies of hyperglycemia and body weight loss. Furthermore, we found treatment with MSC-Exos interferes with STZ-induced inflammation and necroptosis cell death in the heart. Increased IL-10 secretion and increased M2 polarized macrophages in the heart was detected. M2 polarization is induced by cytokine IL-10 and well known to promote tissue repair, participate in wound healing, and reduce inflammation (20, 30). The further detailed presence of monocytes, M1 macrophages, and additional inflammatory cells is needed via flow cytometry to expand the scope of present investigation. These studies will open new avenues for future investigation.

These processes may account for reduced cardiac hypertrophy and fibrosis we observed in MSC-Exos treated diabetic ApoE KO mice. When compared to diabetic mice, treatment with MSC-Exos resulted in reduced pro-inflammatory cytokine expression, and inflammatory TAK1, pJNK, NFKB expression in the heart and an improvement in cardiac function. Additionally, we found increased transcription factor activity of SREBP, LXR, C/EBP, PPAR, COUP-TF, Nur77, and RXR in MSC-Exo treated mice. These transcription factors regulate lipid and carbohydrate metabolism and are heavily dependent on environmental cues like insulin and lipid availability (21–27). As such, we observed activation of these transcription factors reduced in diabetic hyperlipidemic mice. Previous reports have shown diabetes and lipid dysregulation to lead to repressed metabolic regulators like Nur77 expression (38, 39). This downregulation of Nur77 further corresponded to aggravated cardiac dysfunction, fibrosis, enhanced M1 macrophage polarization, and increased NFKB inflammatory pathway expression (38, 39). Moreover, it has been documented that Nur77 and RXR plays a significant role in playing a protective role in diabetes-induced cardiac injury (25, 38, 39).

Chen et al. reported increased expression of the Nur77 regulator leads to decreased Endothelial-to-mesenchymal transition (EndoMT) and reduced transition of cells to fibroblasts that leads to cardiac fibrosis. Mechanistically they showed Nur77 decreases EndoMT by inhibiting the NFKB pathway (39). In the cholesterol transcription factor array we performed (**Supplementary Figure 1**) we detected increased Nur77 transcription factor activity in the MSC-exo treated group when compared to the diabetic ApoE KO. Furthermore, we found significantly decreased expression of NFKB pathway proteins (TAK1, pJNK, NFKB) in MSC-exo treated group when compared to the diabetic ApoE KO mice (**Figure 8**). Our study corresponds with previous studies on MSC-exos, in their ability to significantly reduce fibrosis and hypertrophy in diabetic mice, leading to improved cardiac function parameters like ejection fraction possibly by reduced EndoMT.

Increased activity of LXR, C/EBP, PPAR, COUP-TF, Nur77, and RXR is reported improve diabetic conditions by increasing tissue sensitization to glucose by increasing GLUT4 levels, IRS-AKT signaling, glycolysis, glycogenolysis, and glycerophosphate shuttling (22–27, 40, 41). This indicates that MSC-Exo treatment may also be playing a role in enhancing metabolic regulation which may provide the significant ameliorating effect in diabetic disease progression we observed, further attenuating downstream inflammation and cardiac dysfunction.

In summary, we report novel findings of cardiac damage in diabetic hyperlipidemic mice occurring through inflammation, necroptosis activation, and increased TAK1, pJNK, NFKB expression. Additionally, we provide strong evidence for the use of MSC-Exos in ameliorating this condition which may be beneficial for diabetic patients with dyslipidemia who develop cardiac diseases.

## Data availability statement

The original contributions presented in the study are included in the article/**Supplementary Material**. Further inquiries can be directed to the corresponding author.

## Ethics statement

The animal study was approved by University of Central Florida IACUC Committee. The study was conducted in accordance with the local legislation and institutional requirements.

## Author contributions

AB: Data curation, Formal analysis, Investigation, Methodology, Software, Writing – original draft. DS: Conceptualization, Funding acquisition, Project administration, Resources, Supervision, Validation, Visualization, Writing – review & editing.
