# Supplementary material for: MSC exosomes attenuate sterile inflammation and necroptosis associated with TAK1-pJNK-NFKB mediated cardiomyopathy in diabetic ApoE KO mice
Source: Front Immunol. 2024 Feb 8;15:1348043. doi: 10.3389/fimmu.2024.1348043 (PMC10881775; doi:10.3389/fimmu.2024.1348043)
Supplement: Supplementary file 1 [file DataSheet_1.docx]

Supplementary Material

MSC exosomes attenuate sterile inflammation and necroptosis associated with TAK1-pJNK-NFKB mediated cardiomyopathy in diabetic ApoE KO mice

Abha Banerjee*, Dinender K Singla

*** Correspondence:** Corresponding Author: dinender.singla@ucf.edu

# Supplementary Table 1

| Target | Forward Primer | Reverse Primer |
| --- | --- | --- |
| GAPDH | 5ʹ-ACCCAGAAGACTGTGGATGG-3ʹ | 5ʹ-CACATTGGGGGTAGGAACAC-3ʹ |
| RIPK1 | 5ʹ-GTCATCTAGCGGGAGGTTGG-3ʹ | 5ʹ-CCTCCGCTGTCTAGGTCTGT-3ʹ |
| RIPK3 | 5ʹ-CTCCGTGCCTTGACCTACTG-3ʹ | 5ʹ-CTCACCAGAGGAACCGCATA-3ʹ |
| MLKL | 5ʹ-TGTCTCCCCTGAGAGACTGA-3ʹ | 5ʹ-GTCTCTCCAAGATTCCGTCCA-3ʹ |
| IL33 | 5ʹ-CTACTGCATGAGACTCCGTTCTG-3ʹ | 5ʹ-AGAATCCCGTGGATAGGCAGAG-3ʹ |
| IL1α | 5ʹ-GCAACGGGAAGATTCTGAAG-3ʹ | 5ʹ-TGACAAACTTCTGCCTGACG-3ʹ |

**Supplementary Table 1.** Mouse Specific PCR Primers

# Supplementary Figure 1


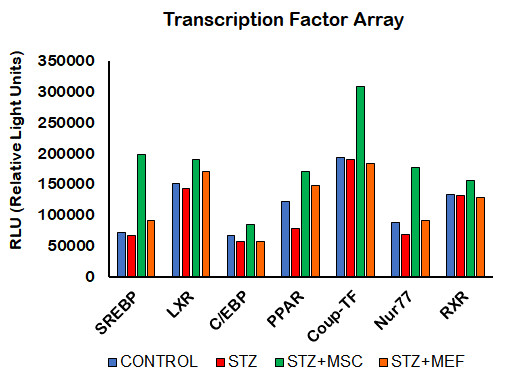


**Supplementary Figure 1.** MSC-Exo promotes reduction of cholesterol metabolism transcription factors SREBP, LXR, C/EBP, PPAR, COUP-TF, Nur77, and RXR; n=2; n = number of animals.
